# Arthroscopic All-Suture Anchor Repair of Medial Meniscus Posterior Root Tears Without a Posteromedial Portal: Clinical Improvement and Healing Despite Persistent Extrusion

**DOI:** 10.3390/jcm14238272

**Published:** 2025-11-21

**Authors:** Murat Aşci, Yavuz Şahbat, Mete Gedikbaş, Utkan Sobay, Fırat Erpala, Taner Güneş

**Affiliations:** 1 Department of Orthopaedics and Traumatology, School of Medicine, Bilecik Seyh Edebali University, 11230 Bilecik, Turkey; muratasci55@gmail.com; 2 Department of Orthopaedics and Traumatology, Bahçeşehir Liv Hospital, 34517 İstanbul, Turkey; yavuzsahbat@gmail.com; 3 Department of Orthopaedics and Traumatology, Kırıkkale Yüksek İhtisas Hospital, 71300 Kırıkkale, Turkey; utkansobay@gmail.com; 4 Department of Orthopaedics and Traumatology, Çeşme Alper Çizgenakat State Hospital, 35950 İzmir, Turkey; drfiraterpala@hotmail.com; 5 Department of Orthopaedics and Traumatology, Acıbadem Eskişehir Hospital, 26130 Eskişehir, Turkey; drtgunes@gmail.com

**Keywords:** meniscus, root tear, repair, suture anchor

## Abstract

**Background:** It is known that meniscus root tears affect the biomechanics of the knee in a way that is equivalent to a total meniscectomy. Therefore, repair is increasingly favored for meniscal root tears. In our study, we aimed to investigate the clinical and radiological outcomes of meniscal root repairs with suture anchors. **Materials and Methods:** Patients who had undergone surgery for medial meniscus posterior root tear (MMPRT) using suture-anchors between 2018 and 2023 were retrospectively analyzed. Patients were excluded if they had a previous infection, a fracture and an operation on the same knee, or osteoarthritis and a follow-up period under one year. The MMPRTs were classified according to the LaPrade classification system. For the functional classification, the range of motion (ROM), the Visual Analog Scale (VAS), the Lysholm Knee Score (LKS), and the International Knee Documentation Committee (IKDC) Subjective Knee Form were used for the postoperative functional assessments. The radiological assessment was performed by measuring the medial meniscus extrusion (MME) and evaluating the signal changes in the magnetic resonance imaging (MRI) of the knee, which was recorded during the last follow-up examination. **Results:** Thirty-two patients (6M/26F) were included in the study. The mean age was 49.9 ± 5.4 years old, and the follow-up period was 29.6 ± 24.1 months. The LKS improved from 53.7 ± 6.9 to 83.6 ± 5.2 and the IKDC improved from 46.1 ± 6.9 to 83.0 ± 5.5 at the final follow-up control (*p* < 0.001 and *p* < 0.001). The VAS score decreased from 8.4 ± 0.5 to 2.5 ± 0.9 (*p* < 0.001). The MRI scan of the knee performed at the last follow-up examination showed no improvement in only one patient. While the MME before surgery was 5.0 ± 2.1 mm, it was 4.6 ± 2.1 mm at the last follow-up examination (*p* = 0.178). An increase in the Kellgren–Lawrence stage was observed in 4 of our patients (from stage 1 to stage 2 in one patient, from stage 0 to stage 1 in 3 patients). **Conclusions:** The results of this study suggest that repairing MMPRTs using suture-anchors is a valid solution for treatment and prevention in patients with poor prognoses in order to achieve positive results in reducing pain, restoring mobility, improving functional outcomes and avoiding a significant increase in progression to arthrosis.

## 1. Introduction

Menisci are essential structures for a healthy knee joint, contributing to stabilization, aiding lubrication, protecting against compressive forces, and enhancing tibiofemoral congruency [1]. Meniscal pathology has been observed in approximately one out of every seven patients presenting to orthopedic clinics with knee-related complaints [2]. Notably, meniscal conditions may present with a variety of etiologies, including degeneration, discoid morphology, and acute meniscal tears. While acute meniscal tears can occur in various configurations within the meniscal body, they can also involve the root attachment sites, with such teras being known as medial meniscal root tears (MMRTs). Compared to tears in the meniscal body or horns, MMRTs are relatively less common and often more challenging to diagnose [3]. As a result, until recently, the pathophysiology and long-term consequences of root tears were not well understood, and such injuries were frequently overlooked, particularly before the widespread availability of magnetic resonance imaging (MRI) [4]. However, the accurate diagnosis and repair of MMRTs are now recognized as critically important, as a substantial proportion of patients undergoing knee arthroplasty have a documented history of untreated meniscal root tears [5].

Meniscal root tears were first described by Pagnani et al. in 1991 as full-thickness radial tears or bony avulsions occurring within 1 cm of the meniscal tibial footprint [6]. These injuries may occur in both acute and chronic settings. The incidence of meniscal root tears demonstrates a bimodal distribution: Acute tears are typically observed in younger individuals following knee hyperflexion injuries, whereas chronic tears are more commonly seen in women over the age of 50 with early to moderate osteoarthritis [4,7,8]. Treatment options for meniscal root tears include non-operative symptomatic management, partial meniscectomy, and repair. Due to the poor outcomes associated with non-operative approaches and partial meniscectomy, practitioners are increasingly favoring meniscal root repair [8]. Among the current surgical techniques, the most commonly employed is the pull-out repair method [8,9]. However, this technique has certain limitations, such as the necessity of creating a tibial tunnel and the risk of suture abrasion and failure due to friction within the tunnel [4,10]. To overcome these drawbacks, all-inside repair techniques utilizing suture anchors have been developed as a promising alternative for meniscal root repair.

The aim of this retrospective study is to evaluate the short- to mid-term clinical outcomes of meniscal root tears repaired using the suture-anchor technique. We hypothesized that meniscal root repair using the suture-anchor technique would result in favorable short- to mid-term outcomes, as reflected by improvements in patient-reported outcome scores (PROs).

## 2. Materials and Methods

### 2.1. Patient Selection and Study Design

Ethical approval for this retrospective study was obtained from the Ethics Committee of Bilecik Seyh Edebali University School of Medicine (Decision No: 291276, Date: 6 November 2024). All patients provided written informed consent prior to their participation. The study was conducted in accordance with the principles of the Declaration of Helsinki.

This study included patients who underwent arthroscopic repair for medial meniscal posterior root tears using the suture-anchor technique between 2018 and 2023. A total of 32 patients between the ages of 18 and 55 years were included. The diagnosis of meniscal root tear was made based on a preoperative MRI and confirmed intraoperatively. Preoperative and intraoperative data were retrieved from the hospital’s electronic medical records and operation notes.

Exclusion criteria included (1) the presence of a rheumatologic disease, (2) a history of knee infection, (3) a previous surgery or fracture in the same knee, (4) radiographically confirmed osteoarthritis classified as Kellgren–Lawrence grade 2 or higher, (5) a hip–knee–ankle (HKA) angle less than 175 degrees resulting in a varus knee, (6) a radiographically confirmed osteochondral lesion classified as Outerbridge grade 3 or higher, (7) an additional meniscus tear or anterior cruciate ligament (ACL) injury, and (8) a follow-up period of less than one year.

### 2.2. Functional and Radiologic Evaluations

Functional evaluations were conducted during the final follow-up visit in January 2025 for all patients with a follow-up duration of at least one year. Two orthopedic and traumatology surgeons (Y.M.A., Ö.C.Ç.), who were not involved in the surgical procedures and were blinded to the patients’ clinical data and treatment outcomes, independently performed both the functional and radiologic assessments. At their final follow-up, patients were evaluated for radiologic findings using 1.5-Tesla MRI.

Patient-reported outcomes were assessed using the visual analog scale (VAS), the Lysholm Knee Score, and the International Knee Documentation Committee (IKDC) Subjective Knee Form [11,12]. Knee range of motion (ROM) was recorded at the final follow-up.

Meniscal root tears were preoperatively classified according to the LaPrade classification system based on MRI findings [13]. The primary radiological outcomes of medial meniscal extrusion (ME) and healing of the meniscus root tissue were evaluated using follow-up knee MRI scans. Additional clinical parameters, such as the presence of postoperative complications (e.g., joint stiffness, persistent pain, or the need for revision surgery), were also documented.

Preoperative and postoperative evaluations of osteoarthrosis were performed using the Kellgren–Lawrence grading system on bilateral knee radiographs. In addition, chondromalacia was assessed according to the Modified Outerbridge classification based on preoperative and final follow-up knee MRI scans.

**Figure 1:**
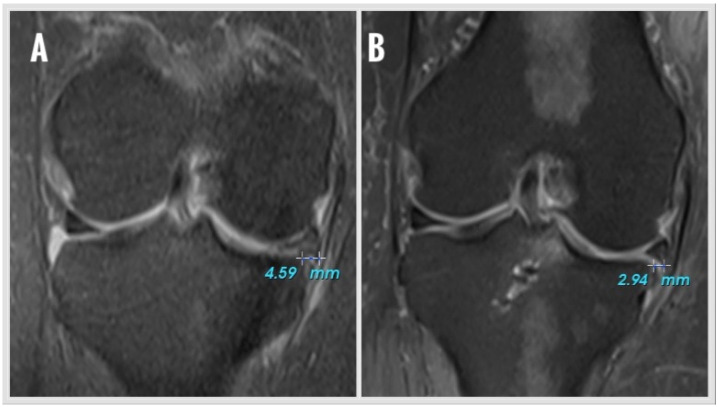
Meniscus extrusion (ME) measurement on coronal MRI section showing the most prominent appearance of the tibial eminence. (**A**) preoperative ME value, (**B**) postoperative ME value.

ME was measured on coronal MRI showing the most prominent appearance of the tibial eminence (Figure 1). A vertical line was drawn from the outer border of the medial tibial plateau, and extrusion was defined as the distance (millimeters) between this line and the outer edge of the medial meniscus. Osteophytes, if present, were excluded from the determination of the tibial plateau margin. The same two blinded observers (Y.M.A., Ö.C.Ç.) performed all the radiologic measurements. Measurements were evaluated intra-inter observer. All intraclass correlation coefficients (ICCs) were above 0.80. The mean of four measurements was used for statistical analysis.

### 2.3. Surgical Technique and Rehabilitation

All procedures were performed under either general or regional anesthesia with the patient positioned supine and a pneumatic tourniquet applied to the proximal thigh. The knee was placed at 90° of flexion throughout the procedure. After routine skin preparation and sterile draping, standard anterolateral (AL) and anteromedial (AM) portals were established, and a comprehensive diagnostic arthroscopy was performed to assess the entire joint, including the patellofemoral articulation and cartilage status.

1. Assessment of Medial Compartment and Access Optimization:

The posterior root of the medial meniscus was carefully probed to confirm the tear configuration. If visualization or instrument access was limited due to a narrow medial compartment, percutaneous pie-crusting of the superficial medial collateral ligament was performed under valgus stress using a 19-gauge spinal needle. This technique increased the working space and allowed for safer manipulation of arthroscopic instruments without causing iatrogenic cartilage damage.

**Figure 2:**
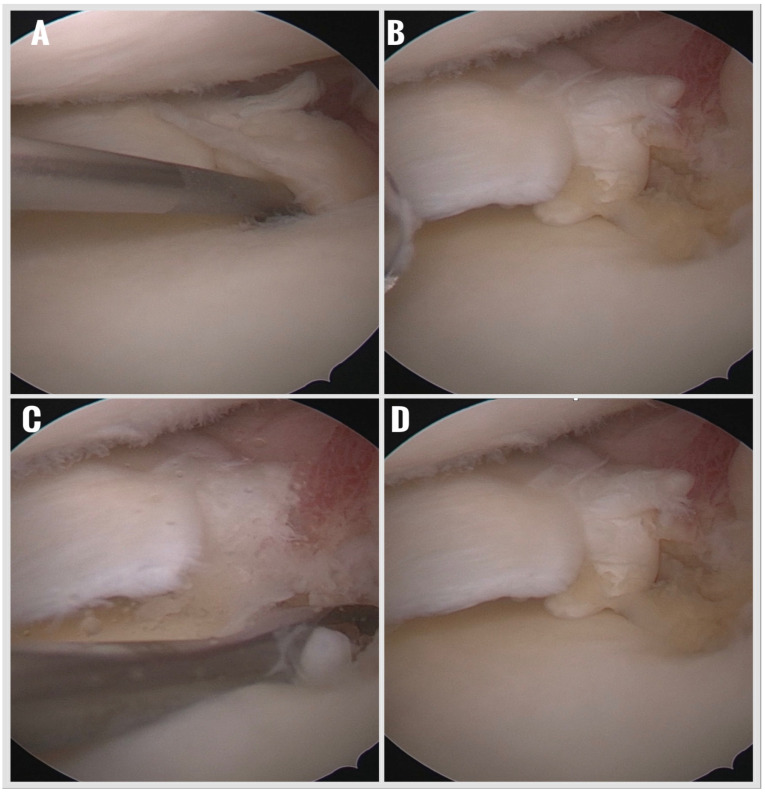
Preparation of torn meniscus and tibial insertion area. (**A**) Detection of a meniscus root tear with an arthroscopic probe. (**B**) Debridement and revitalization of the torn meniscus edges using a shaver. (**C**) Debridement of the meniscus tibial footprint with a curette. (**D**) Prepared state of the footprint and torn menisci. Following surgery, a standardized rehabilitation protocol was initiated.

2. Preparation of the Footprint and Meniscal Tissue:

The torn meniscal root was gently debrided using a motorized shaver to remove frayed tissue and expose healthy edges. The original tibial insertion site was prepared with a curette to remove the fibrocartilaginous layer and expose a bleeding subchondral bone bed, enhancing biological healing potential (Figure 2).

**Figure 3:**
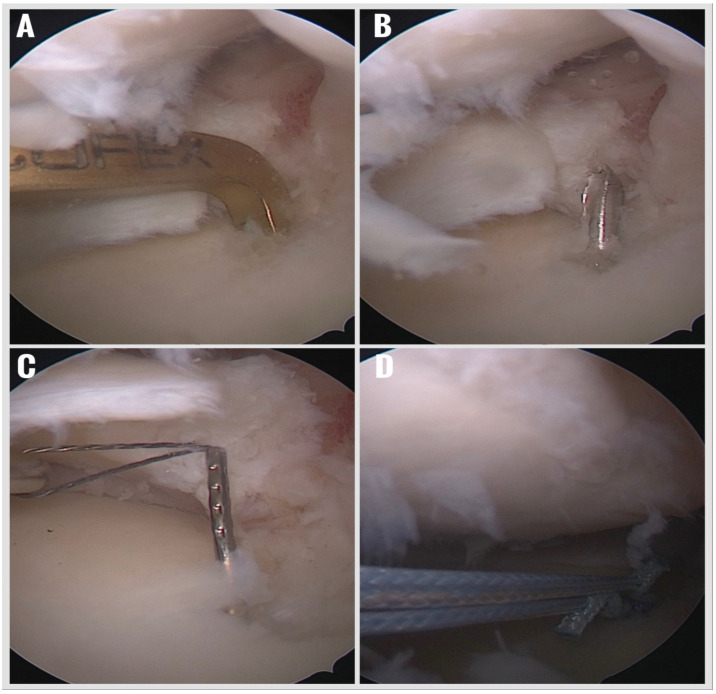
Opening of the tibial tunnel to the previously prepared meniscus attachment area with a 1.8 mm thick K-wire. (**A**) Positioning the K-wire guide. (**B**) Opening the tunnel with the K-wire. (**C**) Pulling the carrier suture into the tunnel with the suture lasso. (**D**) Pulling the suture anchor into the tunnel.

3. Creation of a Retrograde Tibial Tunnel:

With the arthroscope switched to the AM portal for improved visualization, a standard ACL tibial aiming guide was introduced through whichever portal was deemed safest for guide placement—typically the AL portal—and positioned precisely at the anatomic root attachment site. A 1.8 mm Kirschner wire was drilled retrogradely from the anteromedial tibial cortex to the prepared meniscal root footprint, creating a tibial tunnel (Figure 3). A shuttle suture was then passed through this tunnel to facilitate subsequent anchor placement.

4. Retrograde All-Suture Anchor Deployment:

A 1.9 mm all-suture anchor (SutureFix, Smith & Nephew, Andover, MA, USA) was prepared by marking the intended insertion depth and loading it onto a second shuttle suture. The anchor was delivered retrogradely through the tibial tunnel and deployed precisely at the prepared meniscal root footprint. This technique allows for secure fixation while avoiding a tibial tunnel aperture within the joint and eliminating the need for additional posteromedial working portals.

**Figure 4:**
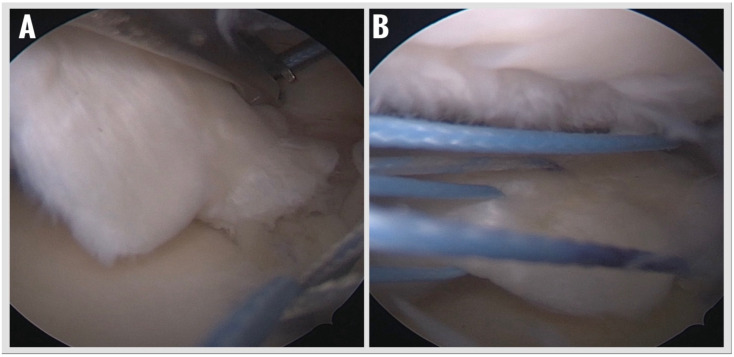
Passing the sutures coming out of the suture anchor through the meniscus using a suture passer to create a modified Mason–Allen configuration. (**A**) Passing sutures through the meniscus with a suture passer. (**B**) Applying the Mason–Allen configuration.

**Figure 5:**
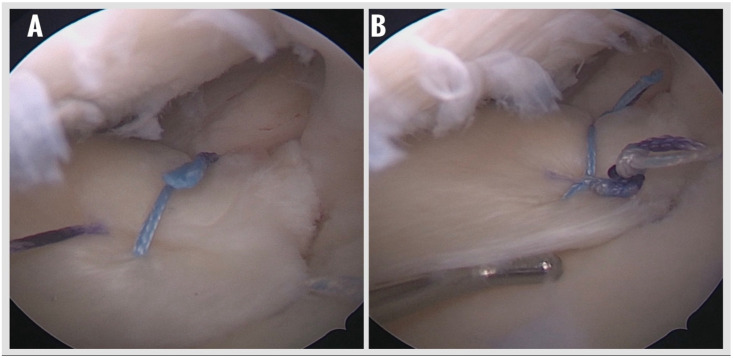
Tying the sutures over the meniscus with appropriate tension and reduction control using a knot pusher. (**A**) Beginning of the knotting of the sutures passing through the meniscus. (**B**) Checking the stability of the meniscus with an arthroscopic probe after completion of the knotting process.

5. Suture Passage and Meniscal Fixation:

The anchor sutures were retrieved into the joint using a suture retriever and then passed through the posterior horn of the medial meniscus using a dedicated meniscal suture passer (Meniscal Scorpion). A modified Mason–Allen configuration was created to optimize tissue capture and distribute load evenly across the meniscal root (Figure 4). Knot tying was performed arthroscopically under direct visualization using a knot pusher to achieve adequate tension and anatomic reduction of the meniscus to its native footprint. Final assessment of fixation stability and tissue tension was performed with a probe (Figure 5).

6. Final Assessment and Closure:

The repair construct was thoroughly inspected for meniscus stability, absence of gapping, and adequate apposition to the tibial footprint. After confirming satisfactory fixation, the portals were closed in the standard fashion, and sterile dressings were applied.

7. Postoperative Rehabilitation:

A standardized rehabilitation protocol was implemented for all patients. Postoperative bracing was not required. Patients were kept non-weight-bearing with crutches for the first six weeks while performing active-assisted range-of-motion exercises and isometric quadriceps and hamstring strengthening. Partial weight bearing was gradually introduced after six weeks, progressing to full weight bearing by eight weeks. Return to unrestricted daily activities was typically permitted at three months, and high-impact sports were restricted until at least six months postoperatively.

### 2.4. Statistical Analysis

Statistical analyses were performed using SPSS Statistics Software (version 23.0, IBM Corp., Armonk, NY, USA). The normality of the data distribution was assessed using the Shapiro–Wilk test. Categorical variables were analyzed using the Pearson Chi-square test, Fisher’s exact test, or the Fisher–Freeman–Halton test, as appropriate. For continuous variables, Student’s *t*-test was used for normally distributed data, while the Mann–Whitney U test was applied for abnormally distributed data. Comparisons of paired non-parametric data (e.g., preoperative vs. postoperative scores) were conducted using the Wilcoxon signed-rank test. A *p*-value of < 0.05 was considered statistically significant for all analyses. Intra- and inter-observer reliability were calculated using the ICC, and an ICC value > 0.75 was evaluated as excellent, 0.75–0.60 as good, 0.59–0.40 as fair, and < 0.40 as poor agreement. Results are reported with 95% confidence intervals (*p* < 0.05).

A pre-study power analysis based on previous data determined a sample size of at least 16 patients to reach the desired power of > 0.8 with 0.67 effect size. The mean postoperative medial meniscal extrusion change was the primary outcome measure for one mean test power analysis [13,14].

## 3. Results

During the study period, a total of 132 patients underwent arthroscopic surgery for meniscal root tears. Of these, 43 patients were treated using the suture anchor technique. After excluding three patients due to having a follow-up duration of less than one year, one patient due to anchor pull-out, five patients with incomplete data, and two who declined participation, the final analysis included a total of 32 patients.

The mean age of the patients was 49.9 ± 5.4 years (range: 37–55), and the mean follow-up duration was 29.6 ± 24.1 months (range: 12–108). The cohort included 26 females (81.2%) and six males (18.8%). Seventeen patients (53.1%) underwent right knee surgery, while 15 patients (46.9%) had surgery on their left knee.

According to the LaPrade classification, four patients (12.5%) had Type I, 18 patients (56.2%) had Type II, nine patients (28.1%) had Type IV, and one patient (3.1%) had Type V meniscal root tears (Table 1). The mean interval from symptom onset to surgery was 11.5 ± 7.8 weeks (range: 1–22). Fifteen patients (46.9%) received physical therapy prior to surgery, and none had documented preoperative intra-articular injections.

Statistically significant and clinically meaningful improvements were observed in all PROs. The Lysholm score increased by a mean of 29.9 points (95% CI, 26.8 to 33.0; *p* < 0.001), and the IKDC subjective score improved by 36.9 points (95% CI, 33.7 to 40.1; *p* < 0.001). Pain severity decreased by 5.9 points on the VAS (95% CI, −6.27 to −5.53; *p* < 0.001). These findings indicate large and precisely estimated functional gains following repair (Table 2).

Follow-up MRI was obtained for all patients. Complete healing was observed in 20 patients (62.5%), among whom eight demonstrated an isointense signal resembling normal meniscal tissue and 12 had an intermediate signal without a high-intensity cleft or ghost sign. Partial healing was observed in 11 patients (34.3%), while one patient (3.1%) showed no signs of healing.

The mean medial ME decreased from 5.0 ± 2.1 mm (range: 2.7–9.0 mm) preoperatively to 4.6 ± 2.1 mm (range: 2.0–9.2 mm) postoperatively. This reduction in extrusion was not statistically significant (*p* = 0.178). At the final follow-up, all patients demonstrated full range of motion in the operated knee.

Based on preoperative and final follow-up knee radiographs, Kellgren–Lawrence staging demonstrated that 16 patients were Stage 0 and 16 were Stage 1 preoperatively. At the final follow-up, 13 patients were Stage 0, 18 were Stage 1, and one patient was Stage II. Three patients who were Stage 0 preoperatively progressed to Stage 1, and only one patient with Stage I advanced to Stage II; however, these changes were not statistically significant (*p* = 0.243). 

According to the modified Outerbridge classification based on MRI findings, no chondral damage was observed preoperatively in 40.6% of patients. At the final follow-up MRI evaluations, progression of the chondral lesion grade was detected in eight patients, and this change was found to be statistically significant (*p* < 0.05) (Table 3).

Three patients were treated for early postoperative infection; two received antibiotic therapy, and one underwent arthroscopic joint debridement. No cases of anchor failure were observed due to infection.

## 4. Discussion

This study’s results demonstrated that meniscal root repair using a suture anchor technique leads to improved short- to mid-term PROs and high healing rates; it does not, however, appear to significantly reduce ME. Meniscal structures are responsible for approximately 40–80% of load transfer. The appropriate repair of meniscal root tears with equivalent effects to total meniscectomy is the accepted treatment approach [15]. More specifically, as the roles of the meniscal structures in knee joint stabilization, lubrication, and load transmission have become better understood, preserving and repairing the meniscus has emerged as the preferred treatment approach [2,8,15,16]. Moreover, the current philosophy in managing all types of meniscal tears emphasizes preservation and repair over resection. For meniscal root tears, treatment options include non-operative management, meniscectomy, high tibial osteotomy (HTO), and repair [17,18,19,20].

Over the past two decades, biomechanical studies have consistently demonstrated that a healed meniscal root following repair provides superior biomechanical properties compared to non-operative management. Currently, non-operative treatment is only considered a viable option for patients with significant comorbidities that make them poor surgical candidates [21,22]. Notably, surgical indications have broadened over time, and there is general consensus that the best outcomes are observed in young, active patients without radiographic osteoarthritis or varus malalignment. While a high body mass index (BMI) and advanced age are not absolute contraindications, they are considered poor prognostic factors. In addition, prolonged symptom duration, Outerbridge grade ≥ 3 cartilage defects, Kellgren–Lawrence grade ≥ 3 osteoarthritis, and varus alignment with a hip–knee–ankle (HKA) angle less than 175 degrees are all associated with unfavorable clinical outcomes. In particular, excessive varus alignment and a high BMI may act synergistically to increase femorotibial contact pressure and reduce healing potential following root repair [23].

In our study, patients with significant varus alignment were excluded, and a combined HTO + root repair was performed in such cases. In line with the previous literature, we observed lower PROs in patients with prolonged symptom duration. Moreover, our careful patient selection, particularly the exclusion of patients with a high BMI, may have contributed to the favorable clinical outcomes observed in this cohort.

Various surgical techniques and their modifications have been described for meniscal root repair. Among these, the two most commonly accepted methods in the literature are the suture anchor repair (SAR) and the transtibial pull-out repair technique [10]. While suture passage and fixation strategies may differ, these two approaches form the foundation of meniscal root repair techniques. Each has distinct technical considerations that may influence a surgeon’s preference. For example, SAR is advantageous in minimizing the bungee effect, but it is technically demanding due to the requirement of a high posteromedial working portal. In the present study, we aimed to eliminate the bungee effect without the need for an additional portal by using a simplified suture anchor technique. This method further avoided potential drawbacks of the pull-out technique, such as micromotion and suture abrasion. Previous studies have shown no significant differences between SAR and pull-out techniques in terms of PROs or healing rates. In our study, the healing rate exceeded 60%, and PROs significantly improved postoperatively. Importantly, these results were achieved without the potential disadvantages associated with either technique. Additionally, in this study, the sutures from the anchor were tied using the modified Mason–Allen stitch configuration, which is known to provide superior primary fixation. Nevertheless, the pull-out technique, particularly when using locked loop sutures, may offer theoretical biomechanical advantages.

In our study, ME did not regress following root repair with the suture anchor technique. Moon et al. investigated the progression of ME and its potential risk factors in patients who underwent medial meniscal root repair using the pull-out technique. They reported that patients with a symptom duration longer than three months prior to surgery showed increased ME at the two-year postoperative follow-up. The authors concluded that early surgical intervention is a key predictor in preventing both the progression of ME and the development of osteoarthritis [21]. This study’s findings are consistent with several studies in the current literature reporting that ME tends to persist regardless of whether suture anchor or transtibial pull-out techniques are used. However, the clinical relevance of persistent extrusion remains unclear, as its correlation with functional outcomes has not yet been definitively established. Despite the limited effect on extrusion, meniscal root repair has been shown to slow the progression of degenerative osteoarthritis. Faucett et al. reported that surgical repair is more cost-effective compared to non-operative management, potentially due to improvements in patient-reported outcomes and a delay in joint degeneration [24].

Also recent evidence suggests that biomechanical and rehabilitative factors may play a crucial role in the persistence or progression of meniscal extrusion following root repair [25,26,27]. Lee et al. demonstrated that radial tear components and osteoarthritis severity were key predictors of medial meniscal extrusion, supporting the notion that persistent displacement can arise from compromised load-sharing mechanisms within the knee [26]. The presence of radial tears in approximately three-quarters of the patients included in our study may also be considered a contributing factor associated with the persistence of meniscal extrusion. Kawada et al. demonstrated that patients with greater postoperative quadriceps muscle strength exhibited significantly less medial meniscal extrusion progression and superior functional outcomes after medial meniscus posterior root repair [27]. These findings imply that insufficient quadriceps recovery and altered load distribution during the postoperative period could contribute to persistent extrusion despite successful anatomic healing. The predominance of female patients in our cohort, along with their relatively low preoperative quadriceps strength and the failure to achieve optimal postoperative muscle recovery, may have contributed to the persistence of meniscal extrusion.

Meniscal root tears are well known to accelerate the progression of osteoarthritis, often leading to total knee arthroplasty (TKA) in the long term [2,28]. Previous reports have shown that more than half of patients treated with meniscectomy alone progress to TKA within 5 years postoperatively, underscoring the importance of anatomic repair in preserving joint integrity [29,30]. In our cohort, although no patients required arthroplasty during the follow-up period, radiological progression consistent with early degenerative changes was observed. Based on the Kellgren–Lawrence classification, three patients progressed from Stage 0 to Stage 1 and one from Stage 1 to Stage II, although these changes were not statistically significant (*p* = 0.243). Similarly, according to the Modified Outerbridge classification, eight patients demonstrated advancement in chondral damage grades, and this change was statistically significant (*p* < 0.05). These findings may suggest that, despite successful functional recovery and pain reduction after suture-anchor root repair, early cartilage deterioration may still occur, emphasizing the need for longer-term follow-up to clarify whether such radiographic changes translate into clinically relevant osteoarthritic progression.

This study has several limitations. First, its retrospective design inherently limited the level of evidence and may have introduced selection bias. Only patients who underwent isolated medial meniscal root repair were included; cases involving combined procedures, such as ACL reconstruction or HTO, were excluded. Moreover, the absence of randomization limits the generalizability and internal validity of the findings. Additionally, the lack of a control group limits the ability to make causal comparisons with other repair techniques, such as pull-out or anchor-based methods. Another important limitation is the lack of second-look arthroscopy, which prevented direct visualization of the meniscal healing process. In addition, although the mean follow-up duration exceeded two years, it may still be relatively short for fully evaluating long-term osteoarthritis progression. Finally, patients with advanced cartilage degeneration or those who underwent concurrent cartilage restoration procedures, such as mosaicplasty, were excluded, which may further restrict the applicability of these findings to more complex cases.

## 5. Conclusions

Meniscal root repair using the suture anchor technique appears promising, as it eliminates the bungee effect and does not require a posteromedial portal. In our cohort, PROs significantly improved at a mean follow-up of three years following surgery. Notably, however, this technique did not result in a significant reduction in ME.

## Figures and Tables

**Table 1:** Demographic data and baseline radiologic data before surgery.

		*n*, %
Gender	Female	26, 81.2%
	Male	6, 18.8%
Laterality	Right	17, 53.1%
	Left	15, 46.9%
LaPrade Classification	1	4, 12.5%
	2	18, 56.3%
	4	9, 28.1%
	5	1, 3.1%
Meniscal extrusion	Positive	13, 40.6%
	Negative	19, 59.4%
Kellgren–Lawrence Classification	0	16, 50%
	1	16, 50%
Outer-Bridge Classification	0	13, 40.6%
	1	7, 21.8%
	2	12, 37.6%

**Table 2:** Preoperative and postoperative clinical scores.

PROs	Preoperative	Postoperative	Mean Change (95% CI)	*p* Value
VAS	8.4 ± 0.56	2.5 ± 0.9	−5.9 (−6.27 to −5.53)	<0.001
Lyscholm	53.7 ± 6.9	83.6 ± 5.2	+29.9 (+26.8 to +33.0)	<0.001
IKDC	46.1 ± 6.9	83.0 ± 5.5	+36.9 (+33.7 to +40.1)	<0.001

**Table 3:** Preoperative and postoperative changes in chondromalasia gradings according to Kellgren–Lawrence and Outerbridge classifications.

Kellgren–Lawrence	Preoperative	Postoperative	*p* Value
0	16	13	0.243
1	16	18	
2	0	1	
**Outerbridge**	**Preoperative**	**Postoperative**	***p* Value**
0	13	11	0.008
1	12	8	
2	7	11	
3	0	2	

## Data Availability

Patient data are available from the corresponding author when requested with a reasonable justification.

## Abbreviations

The following abbreviations are used in this manuscript:

MMRT	Medial meniscal root tear
MRI	Magnetic resonance imaging
ACL	Anterior cruciate ligament
VAS	Visual analog scale
IKDC	International Knee Documentation Committee
ROM	Range of Motion
ME	Meniscal extrusion
ICC	Intraclass correlation coefficient
AL	Anterolateral
AM	Anteromedial
PRO	Patient-reported outcome scores
HTO	High tibial osteotomy
BMI	Body-mass index
HKA	Hip–knee–ankle
SAR	Suture anchor repair
